# 630. Emergence of Colistin Resistance in the OVERCOME Trial: Impact of Combination Therapy with Meropenem

**DOI:** 10.1093/ofid/ofab466.828

**Published:** 2021-12-04

**Authors:** Jason M Pogue, Michael J Rybak, Kyle Stamper, Dror Marchaim, Visanu Thamlikitkul, Yehuda Carmeli, Cheng Chiu, Georgios L Daikos, Sorabh Dhar, Emanuele Durante Mangoni, Achilleas Gikas, Anastasia Kotanidou, Mical Paul, Emmanuel Roilides, Michael Samarkos, Matthew Sims, Dora Tancheva, Sotirios Tsiodras, George Divine, Varduhi Ghazaryan, Keith S Kaye

**Affiliations:** 1 College of Pharmacy, University of Michigan, Ann Arbor, Michigan; 2 Wayne State University / Detroit Medical Center, Detroit, Michigan; 3 Wayne state University, Detroit, MI; 4 Assaf Harofeh (Shamir) medical center, Beer Yaakov, HaMerkaz, Israel; 5 Siriraj Hospital at Mahidol University, Bangkok, Krung Thep, Thailand; 6 Tel Aviv University, Tel Aviv, Tel Aviv, Israel; 7 Chang Gung Memorial Hospital, Taoyuan, Taipei, Taiwan; 8 laiko General Hospital, Athens, Attiki, Greece; 9 John D Dingell VA Medical Center, Detroit, Michigan; 10 Monaldi Hospital, Napoli, Campania, Italy; 11 University Hospital of Heraklion, Heraklion, Iraklion, Greece; 12 Evangelismos Hospital, Athens, Iraklion, Greece; 13 Rambam Health Care Campus, Haifa, HaZafon, Israel; 14 Aristotle University and Hippokration General Hospital, Thessaloniki, Thessaloniki, Greece; 15 Laiko General Hospital, Athens, Lakonia, Greece; 16 Beaumont Health, Royal Oak, Michigan; 17 Emergency Hospital Pirogov, Sofia, Sofiya, Bulgaria; 18 Athens Medical School, National and Kapodistrian University of Athens, Athens, Attiki, Greece; 19 Henry Ford Health System, Detroit, Michigan; 20 NIAID (National Institute of Allergy and Infectious Diseases), Rockville, Maryland; 21 University of Michigan Medical School, Ann Arbor, MI

## Abstract

**Background:**

Colistin (COL) remains an important therapeutic option for carbapenem-resistant (CR) Gram-negative bacilli (GNB). COL is often utilized in combination with meropenem (MEM), in part due to concerns regarding the development of COL resistance with monotherapy. We recently completed a randomized controlled trial comparing outcomes in patients receiving COL + placebo to those receiving COL + MEM; herein we present data on the emergence of COL resistance in this trial.

**Methods:**

OVERCOME was an international, multicenter, randomized, double-blind, placebo-controlled study comparing COL and COL + MEM for the treatment of bloodstream infection and/or pneumonia due to CR GNB. Subjects were included in the modified intent to treat population (mITT) if their enrollment pathogen had a COL MIC ≤2 mg/L, as determined by broth microdilution (BMD). Daily blood and/or respiratory samples were obtained in patients per protocol until two consecutive negatives were obtained or the end of study treatment. All subsequent isolates were evaluated for COL resistance via BMD, defined as MIC ≥ 4 mg/L.

**Results:**

Of the 425 patients in the mITT population, 380 (191 COL; 189 COL + MEM) were evaluable for the endpoint of COL resistance development. The median age of the cohort was 70, 38% were female, 47% were white, and 45% were Asian. 70% had an index infection of pneumonia, 68% were in the intensive care unit at the onset of their infection, and *A. baumannii* was the most common pathogen (78% of patients). Baseline characteristics, infection type, severity of illness, and index pathogen were similar amongst treatment arms. No significant difference in resistance development was seen between the COL and COL + MEM groups overall (12% vs. 8%; p = 0.31), or in any subgroup (Table). In patients with *A. baumannii*, there was a trend towards decreased resistance development with COL + MEM (13.3% vs 7.5%; p = 0.13).

**Conclusion:**

We were unable to identify a significant difference in resistance emergence between treatment arms, but given the low incidence of this outcome, were underpowered to do so. The impact of COL + MEM on preventing emergence of COL resistance in *A. baumannii* warrants further clinical study.

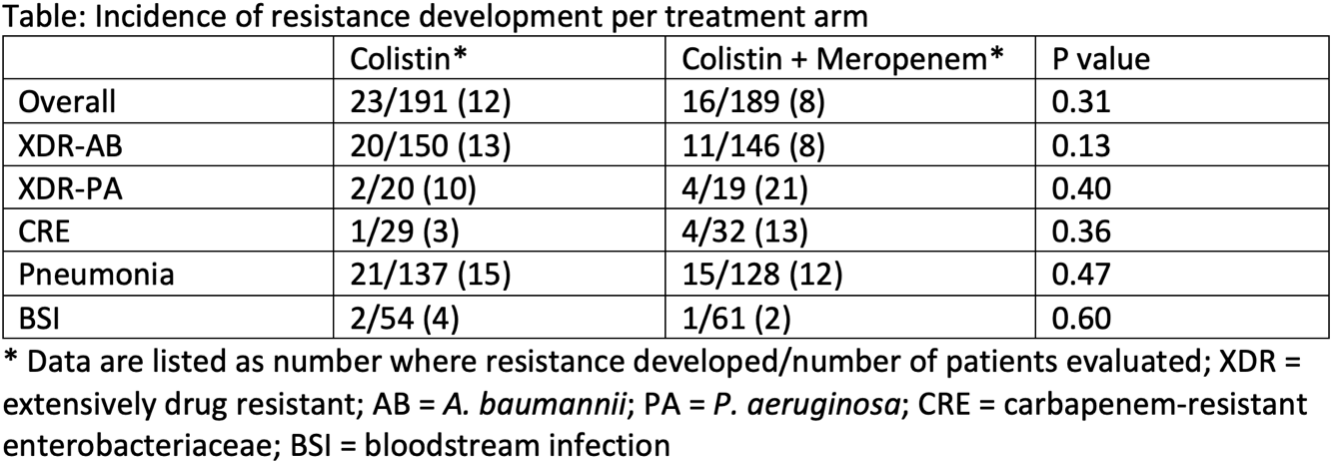

**Disclosures:**

**Jason M Pogue, PharmD, BCPS, BCIDP**, **Merck** (Consultant)**QPex** (Consultant)**Shionogi** (Consultant)**Utility Therapeutics** (Consultant)**VenatoRX** (Consultant) **Michael J. Rybak, PharmD, MPH, PhD**, **Paratek Pharmaceuticals** (Research Grant or Support) **Emmanuel Roilides, MD, PhD, FIDSA, FAAM, FESCMID**, **Merck Sharp & Dohme Corp.** (Consultant, Grant/Research Support) **Matthew Sims, MD, PhD**, **Astra Zeneca** (Independent Contractor)**Diasorin Molecular** (Independent Contractor)**Epigenomics Inc** (Independent Contractor)**Finch** (Independent Contractor)**Genentech** (Independent Contractor)**Janssen Pharmaceuticals NV** (Independent Contractor)**Kinevant Sciences gmBH** (Independent Contractor)**Leonard-Meron Biosciences** (Independent Contractor)**Merck and Co** (Independent Contractor)**OpGen** (Independent Contractor)**Prenosis** (Independent Contractor)**Regeneron Pharmaceuticals Inc** (Independent Contractor)**Seres Therapeutics Inc** (Independent Contractor)**Shire** (Independent Contractor)**Summit Therapeutics** (Independent Contractor)

